# Physical Activity as an Adjunct to Compression Therapy on Healing Outcomes and Recurrence in Patients With Venous Leg Ulcers: A Scoping Review Protocol

**DOI:** 10.3389/fmed.2021.614059

**Published:** 2021-07-08

**Authors:** Yunjing Qiu, Christian Robert Osadnik, Victoria Team, Carolina Dragica Weller

**Affiliations:** ^1^School of Nursing and Midwifery, Monash University, Melbourne, VIC, Australia; ^2^Department of Physiotherapy, Monash University, Melbourne, VIC, Australia

**Keywords:** varicose ulcer, compression therapy, exercise, healing, recurrence

## Abstract

**Background:** Chronic venous leg ulceration is a common and costly clinical issue across the world, affecting up to 3 in 1,000 people. Compression therapy is recommended as the gold standard treatment in clinical practice, although a large number of venous leg ulcers remain unhealed after several years. Physical activity may improve healing although there is limited evidence on the effects of physical activity as an adjuvant treatment to compression to improve venous leg ulcers healing and prevent recurrence.

**Objectives:** This scoping review protocol aims to systematically search, appraise, and synthesize quantitative research evidence to assess the effect of physical activity interventions applied in conjunction with compression therapy on venous leg ulcer healing and recurrence.

**Methods and Analysis:** We will use the methodology framework suggested by Arksey and O'Malley, Levac et al., the JBI as a guide. We will also follow the three-step search strategy recommended by the JBI to systematic search for relevant published research, ongoing clinical trials, and grey literature. Two review authors will independently screen titles and abstracts followed by full-text review to determine final eligibility for inclusion. The search process will be reported using a Preferred Reporting Items for Systematic Reviews and Meta-Analyses flow chart. Characteristics of physical activity interventions, primary outcomes related to ulcer healing and recurrence, and secondary outcomes of interest included quality of life, pain level, adverse effects, and economic costs will be extracted and summarized. The review will provide a descriptive account of the findings from included studies. Where appropriate, data will be pooled for a meta-analysis using a random effects model.

**Discussion:** Physical activity interventions represent a low-cost, potentially useful adjuvant treatment to compression therapy for the management of venous leg ulcers. Several gaps in knowledge remain that are answerable via a targeted scoping review. This protocol outlines the rationale, objectives, and the planned methodology for conducting the study.

**Ethics and Dissemination:** The scoping review will use data from publicly available sources and ethical approval is not required. Findings from this review will be submitted to a peer-reviewed journal, presented at relevant conferences and disseminated via social media.

## Introduction

Venous leg ulcers (VLUs) are the most common type of lower limb ulcers, accounting for around 70% of all types of leg ulcers (1). VLUs usually occur due to malfunction of venous valves that leads to hypertension in the leg veins and damages small blood vessels, which eventually causes a venous leg ulcer to form on the skin especially in the medial aspect of the gaiter area (between knee and ankle) (2). Approximately up to 3 in 1,000 people have an active VLU; and the prevalence increases to 2–4% for people aged 65 and over (3, 4). The prevalence of VLUs is predicted to increase dramatically, due to the growing global population ageing which is projected to grow to 2.1 billion by 2050 (5).

Studies conducted in Western countries reported up to 37% of VLUs reoccur after wound closure within the first 6 months, and up to 48% of VLUs reoccur at 5 years (6). Around one in three patients affected by chronic venous insufficiency (CVI) experience more than 10 episodes of ulceration in a lifetime (7). Between 30–50% VLUs do not heal with compression therapy alone (current best practice treatment): and may remain active for several years (8, 9). The pain, malodorous secretions and slow healing of VLU have been reported to negatively affect patients' physical, psychological, and social functioning (10). These factors, in turn, may impact on patients' willingness to engage in exercise, thus predisposing to further poor outcomes (11).

Management of VLU patients in Australia was estimated to cost the healthcare system ~US$802.55 million (±US307.46 million) per year (12). The costly nature of managing VLUs is also reflected in studies conducted in Sweden (13), the United Kingdom (13), and Germany (14), where the mean cost of treating VLUs ranges from *e*1994 to *e*9569 per patient per year. The average cost of managing an unhealed VLU can be 4.5 times higher than managing a healed VLU (15).

## Study Rationale

Venous incompetence and related venous hypertension are main causes of VLU formation (16). To reduce venous pooling, the efficiency of calf muscle pump is considered as the major mechanism to propel the venous blood from the lower extremities back to heart (17). Smika (18) examined 59 legs with active ulcerations in 48 patients in a specialized leg clinic in Poland to evaluate the prevalence of calf muscle pump dysfunction among patients with chronic venous insufficiency. Calf muscle pump impairment was found in up to 49% of legs, especially the elderly (19). Physical activity, especially that which targets the calf muscle, may increase the effectiveness of the calf-muscle pump and promote venous circulation, thereby increasing ulcer free period for patients (9). For example, Padberg et al. (20) conducted a small randomised controlled trial of 31 participants with CVI in North America to assess the effect of exercise on calf muscle pump function measured as residual volume fraction (RVF) and the mean ejection fraction (EF). The authors reported the mean ejection fraction in exercise group improved from 41.7 to 46.6% (normal values >60%; <40% poor), and the mean RVF improved for the exercise group from 38 to 29.5% (normal value 5–35%) after 6 months of structured leg muscle strength training twice a week (20). This evidence led to progressive resistance exercise being added as a recommendation in the Australian and New Zealand clinical practice guideline for VLU prevention and management (21).

The effect of PA as adjuvant treatment to compression therapy has not, however, been comprehensively addressed. There are proof of concept or mechanistic studies illustrating the potential role of PA as an adjunct to compression, but uncertainty remains regarding its effects on outcomes such as wound healing and recurrence. Three narrative reviews have explored this area, however each focused-on people with chronic venous insufficiency rather than active VLUs (22–24). Knowledge gaps remain regarding the effects of exercise/PA interventions (including those of differing types, frequencies and intensities) and their impacts upon VLUs healing and recurrence. The scoping review aims to address this gap by comprehensively searching, mapping and evaluating the available literature on PA interventions for patients with VLU. The patient experience of undertaking PA in the presence of VLU is another critical area lacking investigation in this field, however this will be specifically examined in a parallel qualitative review being undertaken by the research team.

## Study Objective

The scoping review aims to systematically search, map and identify the evidence regarding the effect of PA interventions applied in conjunction with compression therapy on VLU healing and recurrence. We will describe characteristics of PA interventions (e.g., type, duration, dose intensity), as well as the reported impacts on VLU healing outcomes, calf muscle pump function, quality of life (QoL), pain level, adverse effects, and if available the costs at patient and country level. We will also identify the instruments used to evaluate the pre-defined outcomes of interest.

## Methods and Analysis

The scoping review will be guided by the six-stage framework developed by Arksey and O'Malley (25), further enhanced by Levac et al. (26) and the JBI (27). We will also use the Preferred Reporting Items for Systematic Reviews and Meta-Analyses-ScR extension (28) for scoping reviews checklist to ensure all important sections have been covered.

### Stage 1: Identifying the Research Question

In the review, we aim to answer the following research questions:

- What kinds of adjunct PA/exercise interventions have been used to affect VLU healing and recurrence?
- What methods (i.e., instruments/metrics) have been used to evaluate the key outcomes of interest regarding PA/exercise interventions applied for people with VLU?
- What is the effect of PA/exercise interventions on patient/clinical outcomes (e.g., wound healing, calf muscle pump function, QoL)?

### Stage 2: Identifying Relevant Studies

As suggested by the JBI (27), the search strategy for the review will follow a three-step search to identify both published and unpublished studies. First, an initial search of MEDLINE via OVID will be conducted, followed by an analysis of the keywords contained in the title and abstracts, as well as the subject headings used to describe the study. We will then use all identified keywords and subject headings to undertake search across all included databases. Last, the bibliographies of all identified studies, especially systematic reviews and literature reviews, will be screened for additional studies not identified through electronic database search.

Database search terms will be developed by the research team in conjunction with an experienced research librarian using a combination of keywords and controlled vocabulary (tailored to each database). Electronic database searching will be conducted in Medline, CINAHL and The Cochrane Central from inception to present day, with English language being the only limitation applicable. We will also search ICTRP WHO, ClinicalTrials.gov. and Australian New Zealand Clinical Trials Registry (ANZCTR) for ongoing clinical trials. Grey literature will be retrieved through hand searching the first five pages of google scholar, and the reference list of relevant studies will also be screened for any unpublished studies. A copy of the preliminary search terms and search strategy for Medline (Ovid) is available in Appendix 1.

### Stage 3: Study Selection

The inclusion criteria for the study related to: participants; concepts; context; study type; reported outcomes (29).

#### Participants

We will include studies involving adults (18 years and older) with a clinically diagnosed VLU. Studies reporting outcomes for people with ulcers other than VLU (e.g., arterial ulcers or mixed/ulcers of multiple aetiology) will not be eligible.

#### Concept

The concept examined by the scoping review is PA as an adjuvant treatment to compression for the management of venous leg ulceration. Compression therapy may comprise compression bandages, compression stockings, or any combination of compression therapies. Physical activity refers to any bodily movement produced by skeletal muscles that results in energy expenditure, characterised by frequency, modality, intensity, duration, and context of practice (30). Physical activity is therefore an umbrella term that encompasses both habitual daily activities (e.g., walking, housework) and physical exercise. Exercise is a subset of physical activity that is structured, planned series of body movement aims to improve or maintain physical fitness (31). The precise nature of PA interventions may vary, including recreational activities (e.g., walking, step counts), education programs or structured exercise (e.g., resistance training) or any combination of these. Additionally, PA can be structured or unstructured, supervised or unsupervised.

#### Context

The scoping review will consider studies that have been conducted in any setting, such as home, community, hospital setting, or residential care.

#### Study Types

The scoping review will include experimental and quasi-experimental study designs, such as RCTs, non-equivalent groups design, pre-test and post-test studies, and interrupted time-series studies. Observational studies, including prospective and retrospective cohorts, case-control, cross-sectional studies, as well as case series studies will be eligible for inclusion. Opinion text, oral presentations, conference notes/abstract will be excluded because these types of evidence are not intended to contribute to the measures of effect in PA. Qualitative studies relating to the patients' experience will not be eligible for inclusion as they are the focus of a separate review.

#### Outcomes

Studies will be considered for inclusion, if at least one of the following outcomes are reported:

Primary outcomes:

- Time to healing (as defined by trial authors);
- Proportion of ulcers healed during the trial period (as defined by trial authors);
- Rate of changes in the area of the ulcer during the trial period;
- Incidence of recurrence of healed VLUs.

Secondary outcomes:

- Changes in calf muscle pump function: measured via ejection fraction and residual volume fraction;
- Quality of life: measured using a standardised QoL and well-being questionnaire, including but not limited to ED-5D-5L and SPVU-5D;
- Wound pain measuring via self-reported or other scale;
- Adverse events, such as pain, bleeding, falls, or increased wound exudate;
- Economic outcomes as defined by study author; measured from patient or healthcare perspective.

Studies derived from each included database will be exported to EndNote to remove duplicate records, and then imported to Covidence (32) for screening. Two review authors (YQ and VT/CO) will independently screen citations on titles and abstracts to assess eligibility for full-text retrieval. Citations that are not excluded will be accessed in full-text and eligibility criteria applied to determine a final inclusion yield. We will record all reasons for full-text exclusion and represent in a PRISMA (33) flow diagram that were adopted for scoping review as recommend by the JBI guideline (Appendix 2). Any disagreements related to the study inclusion and exclusion will be resolved by discussion or a third independent assessor if necessary. Where the eligibility of a study is unclear, we will attempt to contact authors for clarification.

### Stage 4: Charting the Data

A data extraction table will be designed by two independent reviewers (YQ, VT) to collect key information for data synthesis. A proposed data extraction table is presented in Appendix 3. As suggested by Levac et al. (26), two independent reviewers (YQ, VT) will independently extract information from the first 10 studies to ensure their extraction approach is consistent with research questions, and form may be further refined accordingly. We will extract the following data from the included studies if applicable (25, 27):

- Author(s), year of publication, country;
- Study design, study setting;
- Study size;
- Outcomes;
- Main findings;
- Funding source.

As per the Consensus on Exercise Reporting Template (CERT) tool, we will report the following characteristics of PA interventions where available (34):

- The type of exercise equipment used
- Who provided the training
- How and when the intervention was delivered to patients
- Location of intervention delivery
- Dose intensity of the intervention.

### Stage 5: Collating, Summarizing, and Reporting Results

The scoping review will provide a descriptive account of the findings extracted from included studies. We will group study findings according to PA type and intensity to allow for exploration of potential dose response effects. The detailed information about reported PA intervention, the primary, and secondary outcomes as listed above will be extracted from each study. Tabulations will be the principal method of summarising data, however opportunities to pool via meta-analysis (e.g., comparable outcome data across multiple RCTs) will be sought where possible, adopting a random effects model.

### Stage 6: Consultation

Consultation with patients, carers, and health professionals is an opportunity to share experiences of VLU treatment and management outcomes to find out what is working well and how we, as researchers, can improve VLU management in the future. The consultation steps will be carried out with patients, carers and health professionals as part of an established external wound consumer advisory group. The wound consumer advisory group was established in partnership with patients and family carers to better understand the patient perspective. The first interview will be held at the start of the scoping review to explore patient needs about PA in VLU healing and recurrence in the scoping review. Patient input may give rise to altered (i.e., additional) scoping review aims, which will be added to the study aims we have documented. The second consultation will be conducted at the end of the scoping review. We will share the review findings with the wound consumer advisory group to identify any additional emerging issues relating to PA as an adjuvant therapy to VLU management not identified in the scoping review. The findings of the scoping review and the comments from the consultation will inform the intervention for a future pilot RCT to investigate the impact of PA on VLU healing and recurrence.

## Discussion

This protocol outlines the rationale, objectives, and planned methodology for conducting a scoping review to assess the effect of PA interventions as adjuvant therapy on VLU healing and recurrence. The methodology framework suggested by Arksey and O'Malley (25), Levac et al. (26), and the JBI (27) will be used as a guide to enhance transparency and accuracy. The review will describe the characteristics of PA interventions and their reported impact on clinically important VLU healing outcomes, as well as identify the instruments used to evaluate the pre-defined outcomes of interest. The findings will establish a clear evidence base for future research and help guide healthcare practice regarding PA for the management of VLU.

## Ethics Statement

This scoping review will collect data from publicly available resources and the consultation process will be held as a public consultation process. Ethics approval will therefore not be required. The scoping review article will be submitted to a peer-reviewed journal, disseminated at relevant national and international conferences and communicated to the scientific and public community via other channels (e.g., social media).

## Author Contributions

CW devised the project and the main conceptual ideas. YQ worked the experienced librarian (CF) to develop search strategies and wrote the manuscript with support from CO, VT, and CW. CO reviewed and modified the search strategies. All authors contributed to the design and the frameworks for this protocol, provided critical feedback, reviewed, and approved the final manuscript.

## Conflict of Interest

The authors declare that the research was conducted in the absence of any commercial or financial relationships that could be construed as a potential conflict of interest. The handling Editor declared a past co-authorship with several of the authors VT, CW.
