# Supplementary material for: Physical Activity as an Adjunct to Compression Therapy on Healing Outcomes and Recurrence in Patients With Venous Leg Ulcers: A Scoping Review Protocol
Source: Front Med (Lausanne). 2021 Jul 8;8:614059. doi: 10.3389/fmed.2021.614059 (PMC8295537; doi:10.3389/fmed.2021.614059)
Supplement: Supplementary file 1 [file Data_Sheet_1.docx]

**Appendix 1: search strategy for MEDLINE**

Searching strategy for Ovid MEDLINE

| **#** | **Searches** |
| --- | --- |
| 1 | Varicose ulcer/ or leg ulcer/ |
| 2 | ((varicose or leg or venous or stasis or lower or extremit* or crural or ulcus) adj2 ulcer*).mp. |
| 3 | Ulcus cruris |
| 4 | 1 or 2 or 3 |
| 5 | Rehabilitation/or Cardiac Rehabilitation/ |
| 6 | "Activities of Daily Living"/ |
| 7 | Early Ambulation/ |
| 8 | Recreation therapy/ or telerehabilitation/ or physical therapy modalities/ |
| 9 | exp walking/ |
| 10 | exp exercise/ |
| 11 | exp exercise movement techniques/ |
| 12 | (exerci* or rehabilitat* or physiotherap* or walk* or physical activit* or calf raise* or heel raise* or calf muscle pump*).mp. |
| 13 | ((strength* or endurance*) adj2 train*).mp. |
| 14 | 5 or 6 or 7 or 8 or 9 or 10 or 11 or 12 or 13 |
| 15 | 4 and 14 |
| 16 | Limit 15 to English language |

**Appendix 2**

Records after duplicates removed
(n = )

Additional records identified through other sources
(n = )

Records identified through database searching
(n = )

Records excluded
(n = )

Records screened
(n = )

Full-text articles assessed for eligibility
(n = )

Full-text articles excluded, with reasons
(n = )

Studies included in quantitative synthesis
(n = )

Figure 1. PRISMA flow diagram, adapted from Moher et al. (2009)

**Appendix 3**

*Description of included studies*

| **Author (s),**  **Year,**  **Country** | **Study**  **design** | **Study settings** | **Study size** | **PA interventions**  **characteristics** | **Outcome measures** | **Main findings** | **Funding source** |
| --- | --- | --- | --- | --- | --- | --- | --- |
|  |  |  |  |  |  |  |  |
|  |  |  |  |  |  |  |  |
|  |  |  |  |  |  |  |  |
